# Spatially Explicit Landscape-Level Ecological Risks Induced by Land Use and Land Cover Change in a National Ecologically Representative Region in China

**DOI:** 10.3390/ijerph121114192

**Published:** 2015-11-09

**Authors:** Jian Gong, Jianxin Yang, Wenwu Tang

**Affiliations:** 1 Department of Land Resource Management, School of Public Administration, China University of Geosciences (Wuhan), 388 Lumo Road, Hongshan District, Wuhan 430074, China; E-Mails: gongjian@cug.edu.cn (J.G.); mr.yangjx@gmail.com (J.Y.); 2 Key Labs of Law Evaluation of Ministry of Land and Resources of China, 388 Lumo Road, Hongshan District, Wuhan 430074, China; 3 Department of Geography and Earth Sciences, The University of North Carolina at Charlotte, 9201 University City Blvd., Charlotte, NC 28223, USA; 4 Center for Applied Geographic Information Science, The University of North Carolina at Charlotte, 9201 University City Blvd., Charlotte, NC 28223, USA

**Keywords:** land use and land cover change, landscape ecological risks, ecologically representative region, spatiotemporal simulation

## Abstract

Land use and land cover change is driven by multiple influential factors from environmental and social dimensions in a land system. Land use practices of human decision-makers modify the landscape of the land system, possibly leading to landscape fragmentation, biodiversity loss, or environmental pollution—severe environmental or ecological impacts. While landscape-level ecological risk assessment supports the evaluation of these impacts, investigations on how these ecological risks induced by land use practices change over space and time in response to alternative policy intervention remain inadequate. In this article, we conducted spatially explicit landscape ecological risk analysis in Ezhou City, China. Our study area is a national ecologically representative region experiencing drastic land use and land cover change, and is regulated by multiple policies represented by farmland protection, ecological conservation, and urban development. We employed landscape metrics to consider the influence of potential landscape-level disturbance for the evaluation of landscape ecological risks. Using spatiotemporal simulation, we designed scenarios to examine spatiotemporal patterns in landscape ecological risks in response to policy intervention. Our study demonstrated that spatially explicit landscape ecological risk analysis combined with simulation-driven scenario analysis is of particular importance for guiding the sustainable development of ecologically vulnerable land systems.

## 1. Introduction

The objective of this study is to investigate land use and land cover change (LULCC) and associated landscape-level ecological risks in a national ecologically representative region, Ezhou City, China. LULCC, is an important theme in the study of social-ecological systems [1,2,3], and is driven by a range of factors from environmental, socio-economic, and political domains [4,5,6]. The influence of these interacting factors drives human decision-makers to modify (e.g., via urban expansion) the landscape of the land system in which they are situated. Thus, heterogeneous and dynamic landscapes are generated from these human activities, which further produce a series of environmental, ecological, and social impacts on land systems of interest. Landscape patterns that human actors interact with, represent the spatial consequence of underlying biophysical or socioeconomic processes. Change in landscape patterns due to LULCC is a form of anthropogenic stressor and may lead to, for example, biodiversity loss, habitat fragmentation, and environmental pollution [4,6]. In particular, in those regions that are ecologically vulnerable or representative, the environmental or ecological effects of LULCC are often severe or adverse, contributing to a series of public health issues (e.g., air- or water-borne diseases due to dust or contaminants from pesticides or fertilizers) [7,8]. Thus, the evaluation of the environmental or ecological effects of LULCC on these ecologically representative regions is of critical importance for the development of ecological security and sustainability of these regions.

The analysis or assessment of landscape-level ecological risks (landscape ecological risks hereafter) offers a means of supporting the evaluation of ecological or environmental effects of LULCC. Landscape ecological risk has gained considerable attention from researchers interested in the study of LULCC and related domains [9,10,11,12,13]. In general, ecological risk is a reflection of the possibility that an ecosystem maintains itself in a low-energy equilibrium with relatively stable structures and functions in response to disturbances from external factors [14,15]. The value of ecological risk is associated with the degree of the external disturbance and the ecosystem’s internal capability (*i.e.*, vulnerability and sensitivity) to cope with this form of disturbance. Landscape patterns are the outcome of the interactions between physical environments and human activities that may create alternative disturbances [16]. Change in landscape patterns has substantial influence on the flow of materials and energy, and habitat or environmental quality. Thus, landscape pattern change is linked to the internal process of an ecosystem and can then be regarded as a direct reflection of ecological risk at regional or landscape level. Graham *et al.* [9] stressed that the analysis of ecological risks at a regional level must take into account spatial characteristics of the landscape for an ecosystem of interest. Norton *et al.* [11] presented a generic framework used by the U.S. Environmental Protection Agency (EPA) to support the assessment of ecological risks. This framework suggested three phases for ecological risk assessment: Problem formulation (including identification of key driving factors), characterization of exposure and ecological effects, and risk characterization. This three-phase framework provides effective guidance for subsequent ecological risk assessment efforts [14,15,17,18,19].

Benefiting from advances in landscape ecology and ecological modeling [16,20], landscape metrics have been developed to support the quantification of landscape patterns with respect to composition and configuration [21,22]. These landscape metrics allow for evaluating the spatially explicit characteristics of landscape patterns and associated change [16,23]. Therefore, these landscape metrics have been used as a critical component to help evaluate landscape ecological risks of a land system of interest [9,12,13]. For example, Graham *et al.* [9] identified a set of spatially explicit endpoint measures (*i.e.*, driving factors), including two landscape measures (dominance and contagion), for their landscape ecological risk assessment in a forest-dominated landscape exposed by ozone concentration. Xie *et al.* [12] studied the spatial distribution of ecological risks based on a combined use of landscape disturbance and vulnerability indices in the Poyang Lake region, China. The landscape disturbance index used by Xie *et al.* was a function of landscape metrics. Likewise, Peng *et al.* [13] proposed a similar landscape ecological risk and applied this index to evaluate the adverse effect of mining-centric land use practices at the watershed level. While these studies reported in the literature show the significance of landscape metrics in evaluating ecological risks, investigations on how landscape ecological risks change over time under policy intervention and its possible futures remain inadequate. This investigation is, however, fundamentally important for those ecologically vulnerable regions, for example, in China.

In this study, we therefore focus on the evaluation of historic and future landscape ecological risks induced from LULCC in Ezhou City, China. Ezhou City is a national level ecologically representative region located in the middle course of Yangtze River. This region has experienced rapid LULCC in the past few decades and is under the influence of alternative government policies for farmland protection, ecological conservation, and urban planning. For example, farmland protection policies are represented by the “requisition-compensation balance” national policy that dates from 1997, which requires the reclamation of farmlands (from other land cover types) with the same quantity and quality as those that are occupied [24]. The “Grain for Green” national program for ecological conservation was initiated in 1999 to encourage the conversion of low-quality farmlands to forests [25]. Further, Ezhou City has developed its own local urban planning policy to serve the needs of increasing urbanization. Land use conflicts thus often exist when taking into account these policies serving for different purposes. The study of landscape ecological risk and its potential future alternatives in response to policy intervention in this region will provide substantial support for the resolution of land use conflicts and sustainable development of land systems in the region. It will also offer valuable insights into, for example, the early warning of adverse environmental or ecological effects induced by LULCC. Thus, in this article, we conducted analyses on the spatiotemporal characteristics of LULCC by combining Geographic Information Systems (GIS; see [26]) and landscape pattern analysis. We carried out the evaluation of landscape ecological risks of LULCC in our study region. With support from a spatiotemporal simulation model, we conducted a scenario analysis of alternative policy intervention and evaluated its potential impacts on future landscape ecological risks in this national ecologically representative region.

## 2. Materials and Methods

### 2.1. Study Area and Data

**Figure 1:**
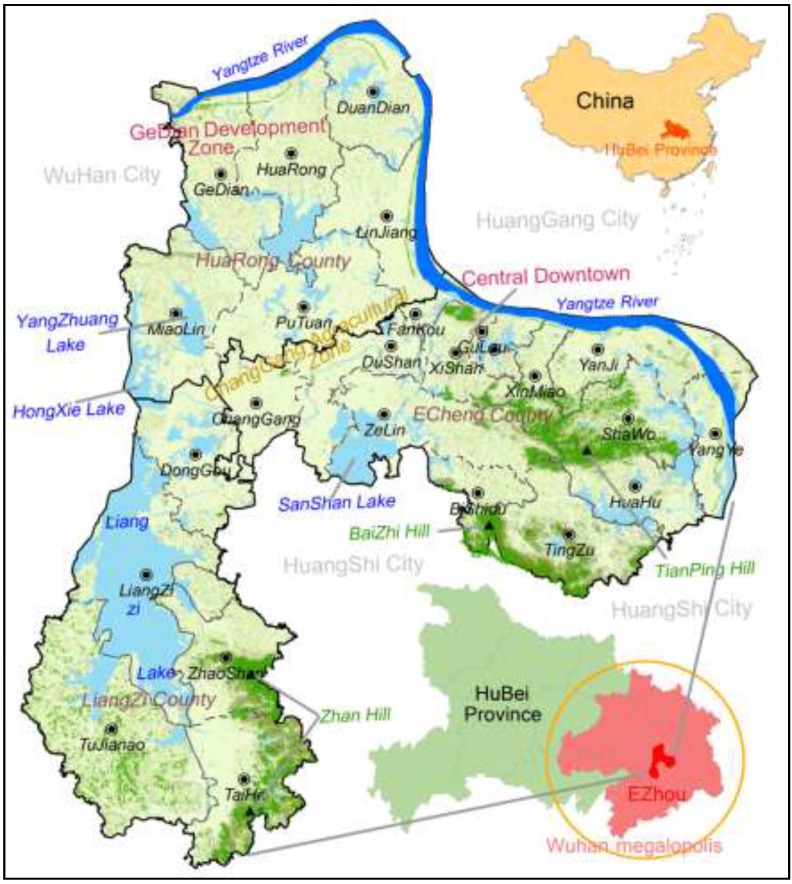
Map of the study area: Ezhou City, China.

**Figure 2:**
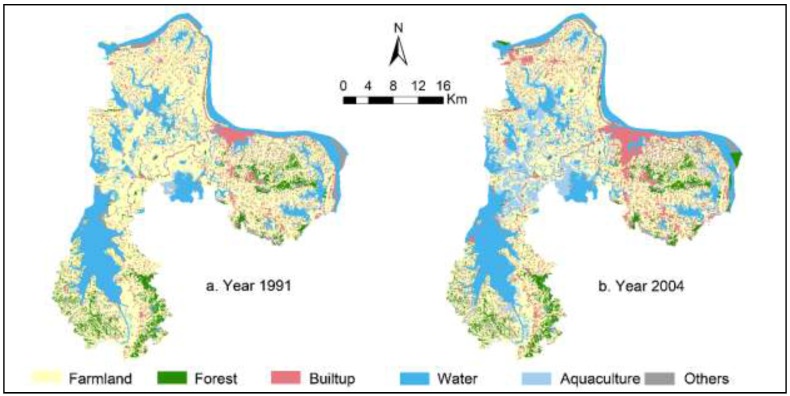
Maps of land cover patterns of Ezhou City in 1991 and 2004.

Ezhou City (see Figure 1) is located in the southeastern region of Hubei province, China (114°32′E–115°05′E, 30°00′N–30°06′N). Ezhou City belongs to the Greater Wuhan Metropolitan region and consists of three counties (Echeng, HuaRong, and LiangZi Lake) encompassing 25 towns. Ezhou City, covering 1596 km^2^ in total, is dominated by undulating topography (high in the southeast, and low in northwest and middle) and diverse geomorphology: Alluvial terrace in the north, foothills in the east and southeast, plains in the northwest and southwest, and lacustrine plains in the middle. The study area is characterized by a humid subtropical climate with typical monsoon seasons (hot summers and cold winters). Soil types in Ezhou City include Quaternary Period brown red soil, yellow brown soil, and grey soil. Ezhou City is also known as the City of a Hundred Lakes, with a 90-km streamline of Yangtze River and 20,973 ha of lake area (6 large, 20 medium-sized, and 129 small lakes). Ezhou City has been playing a critical role in ensuring regional-level ecological security due to its rich biodiversity and water resources. Ezhou City has been established as a national lake conservation region and a ecologically representative region at the national level. The study region has experienced substantial LULCC since 1979 (see Figure 2 for maps of land cover patterns in 1991 and 2004). This rapid LULCC has led to severe water and air pollution in this region due to, for example, unregulated or over use of pesticides and fertilizers for aquaculture and agriculture, and discharge of untreated wastewater [27,28].

We obtained land cover data from 1991, 2004, and 2013 for our study region from the Department of Land and Resources Administration, Ezhou City. These land cover data were developed through remote sensing classification (from aerial imagery and SPOT 5 satellite imagery) and field validation (classification accuracy: 93%–95%) [29]. All of the land cover data were organized and re-projected into a single coordinate system. We further reclassified land cover data into six classes: farmland, forests, built-up land, water bodies, aquaculture, and other lands (including orchard, rangeland, wetlands, and other open space). Elevation data are at a 30 m × 30 m spatial resolution from the Geospatial Data Cloud from the Chinese Academy of Science (see http://www.gscloud.cn). Other geographic information system (GIS) datasets, including highways, railways, roads, stream networks, and jurisdictional boundaries were provided by the Department of Urban-Rural Planning, Ezhou City (cartographic scale: 1:200,000). Spatial data of basic farmland protection zones and ecological conservation zones were obtained from the Department of Land and Resources Administration, Ezhou (cartographic scale: 1:10,000).

### 2.2. Methods

**Figure 3:**
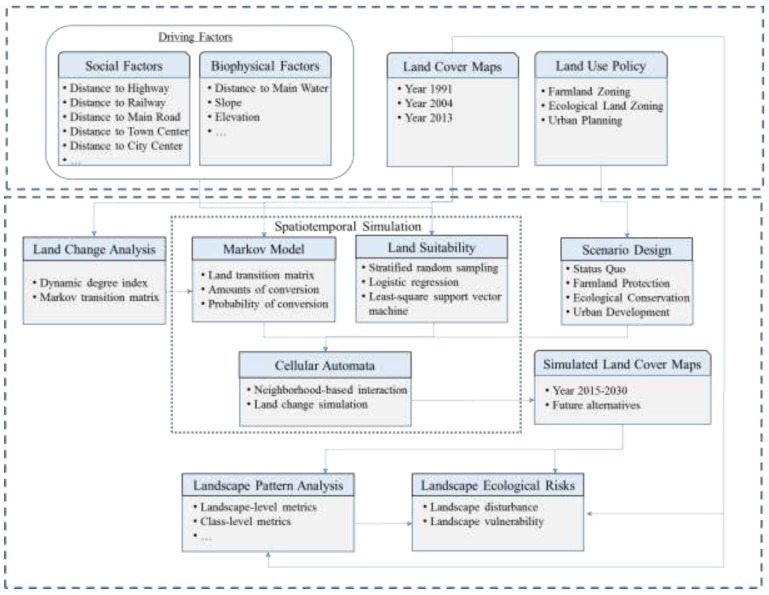
Spatially explicit modeling framework of land use and land cover change and associated landscape ecological risks.

We present a spatially explicit modeling framework that integrates a set of indices and models to enable the evaluation of spatially explicit landscape ecological risks (see Figure 3). Specifically, this framework consists of five key components: Land change analysis using dynamic degree index and Markov transition matrix, landscape pattern analysis using landscape metrics, landscape ecological risk analysis, spatiotemporal simulation of LULCC, and scenario analysis.

#### 2.2.1. Land Change Analysis Using Dynamic Degree Index and Markov Transition Matrix

To evaluate the dynamics of land cover change in our study area, we chose to use dynamic degree index [30]. Dynamic degree index (also referred to as ratio of change or land change index) reflects the magnitude of land cover change and potential hotspots. Dynamic degree index has a focus on the process of land cover change instead of the outcome. The dynamic degree index of land change within a specific time period is calculated as follows:

(1) $$LC=\left(\frac{\sum_{i=1}^{n}\Delta LU_{i\to j}}{\sum_{i=1}^{n}LU_{i}}\right)\times 100\text{\%}$$

where *LC* is the dynamic degree index that represents change ratio of land conversion. $\Delta LU_{i\to j}$ denotes the area of land cover changing from type *i* to type *j*. $LU_{i}$ is the area of land cover type *i*, and *n* is the number of land cover types.

We also used a Markov transition matrix approach (see [31,32]) to evaluate change among land cover types. A Markov transition matrix records the amount of land converted between land cover types. From this matrix, we can further derive the transition probability of a specific land conversion type between two time periods.

#### 2.2.2. Landscape Pattern Analysis Using Landscape Metrics

Landscape metrics have been extensively used to quantify characteristics of landscape patterns [16,33,34]. In this study, we chose three types of landscape metrics to quantitatively evaluate landscape characteristics (see [33,34] for detail): (1) landscape fragmentation: Splitting index (SPLIT), patch density (PD), and contagion (CONTAG); (2) geometric features: Perimeter-area fractal dimension; and (3) landscape diversity: Shannon’s diversity index. While all of these metrics at the landscape level are considered, splitting index, patch density, and perimeter-area fractal dimension at the class level are also derived. These landscape metrics allow us to evaluate the impact of natural and human drivers on landscape patterns and associated structural characteristics. Spatiotemporal patterns of these landscape metrics are helpful for evaluating landscape characteristics and associated spatiotemporal heterogeneity. The software that we used to derive landscape metrics is FRAGSTATS version 4 [35].

#### 2.2.3. Landscape Ecological Risk Analysis

Ecological risk is a reflection of the possibility that an ecosystem maintains itself into a low-energy equilibrium with relatively simple structures and functions in response to disturbance from external factors. The value of ecological risk is associated with the degree of the external disturbance and its vulnerability *per se*. In this study the metric of ecological risk that we used is based on the combination of two types of landscape-level indices: landscape disturbance index (external) and landscape vulnerability index (internal) (see [12,13]). Landscape disturbance index measures the magnitude of the disturbance from natural and human drivers at the landscape level, which can be represented using a function of a suite of landscape metrics. The landscape disturbance index used in this study is a weighted function of three landscape metrics: splitting index, landscape fragmentation, and landscape diversity. Landscape disturbance index for a specific land cover type is calculated as follows:

(2) $$D_{i}=w_{1}\times SPLIT_{i}+w_{2}\times PD_{i}+w_{3}\times SHDI$$

where *w_1_*, *w_2_*, *w_3_* represent the weights of each metric. As suggested in the literature (see [12]), *w_1_* = 0.3, *w_2_* = 0.5, and *w_3_* = 0.2 in this study. *i* corresponds to land cover type *i*. Landscape metrics are normalized before calculating landscape disturbance index. In our study area, landscape disturbance from human drivers (e.g., road construction, impervious surface) are much greater than that from natural drivers. This leads to the degradation of natural landscape functions, worsening the natural habitat in our study area.

Landscape vulnerability index evaluates the internal capability of a land cover type to maintain its current structure and function (similar to ecological succession and stability; see [16]). Landscapes are different in terms of species richness, characteristics of material and energy flows, and ability to respond to external disturbance. Land cover types with high landscape vulnerability are characterized by high risk of structural change and function loss in the face of external disturbance. Natural land cover types (e.g., wetland or barren lands) often have high vulnerability since their structures and functions are sensitive to external disturbance. In contrast, those land cover types with intensive management efforts from human beings (for example, built-up lands) have high stability with respect to structure and function to resist external disturbance (low vulnerability). Further, most of the forests in our study area are man-made (shelter or commercial forests) instead of natural—*i.e.*, their vulnerability is relatively low due to continual land management practices. Thus, we organized land cover types in our study area into six grades of vulnerability: Built-up land (grade 1; lowest), forest land (grade 2), farmland (grade 3), aquaculture land (grade 4), water bodies (grade 5), and other land (grade 6; highest). In this study, the vulnerability index for a specific land cover type (noted as *V_i_*) is obtained after normalization on the grades of vulnerability.

Thus, the index of landscape ecological risk is represented as a weighted sum function of landscape disturbance index and landscape vulnerability index. The index of landscape ecological risk is estimated as follows:

(3) $$R=\overset{n}{\underset{i=1}{\sum }}\alpha_{i}*{D_{i}}^{1/2}*{V_{i}}^{1/2}$$

where *R* denotes the index of landscape ecological risk for a specific analysis region of interest. $\alpha_{i}$ represents the weight on a specific land cover type (usually calculated as the ratio of the area of the land cover type over the total area of analysis regions), *D_i_* is the landscape disturbance index, and *V_i_* is the landscape vulnerability index for land cover type *i* in the analysis region.

Based on the landscape ecological risk model in this study, we calculated ecological risks for our study area and each of its towns to analyze the spatiotemporal patterns of ecological risks. We applied the following criteria to evaluate ecological risks using five categories: Low (0–0.2), relatively low (0.2–0.4), medium (0.4–0.6), relatively high (0.6–0.8), and high (0.8–1.0).

#### 2.2.4. Spatiotemporal Simulation Based on Cellular Automata Coupled with Markov Model

To generate future alternatives of land cover patterns for projected ecological risk analysis, we used a spatiotemporal simulation approach based on the coupling of cellular automata (CA) and Markov model—also known as Markov-CA model [36,37,38]. While Markov model supports the determination of amounts and transition probabilities of land conversion, CA enables the simulation of where and how land conversions occur. CA is a bottom-up simulation approach that is based on neighborhood interactions to guide the state transition of a spatial system of interest [39,40]. The landscape of the system is rasterized into a lattice of cells that interact with their neighbors using rules. Neighborhood interactions in a CA may drive the emergence of complex spatial patterns at the macro level. Thus, CA has a wide variety of applications in the simulation of complex spatial phenomena [39,41,42,43] including urban sprawl, land use and land cover change, and transportation planning. A typical CA model is composed of four components [39]: Cellular automata, the spatial dimension of these automata, neighborhood, and transition rules. In particular, transition rules are key to the use of CA for the simulation of complex spatial phenomena. Transition rules can be calibrated through logistic regression and multi-criteria evaluation [39,42], which are typically linear approaches. However, these linear approaches may not be suitable for capturing the nonlinearity of land use and land cover change. Thus, in this study, we used a Least Square Support Vector Machine (LSSVM; see [44]) as a nonlinear regression approach for the generation of transition rules of CA. Each type of land conversion corresponds to a LSSVM model—in total, 36 LSSVM were used to cover all land transition types.

**Figure 4:**
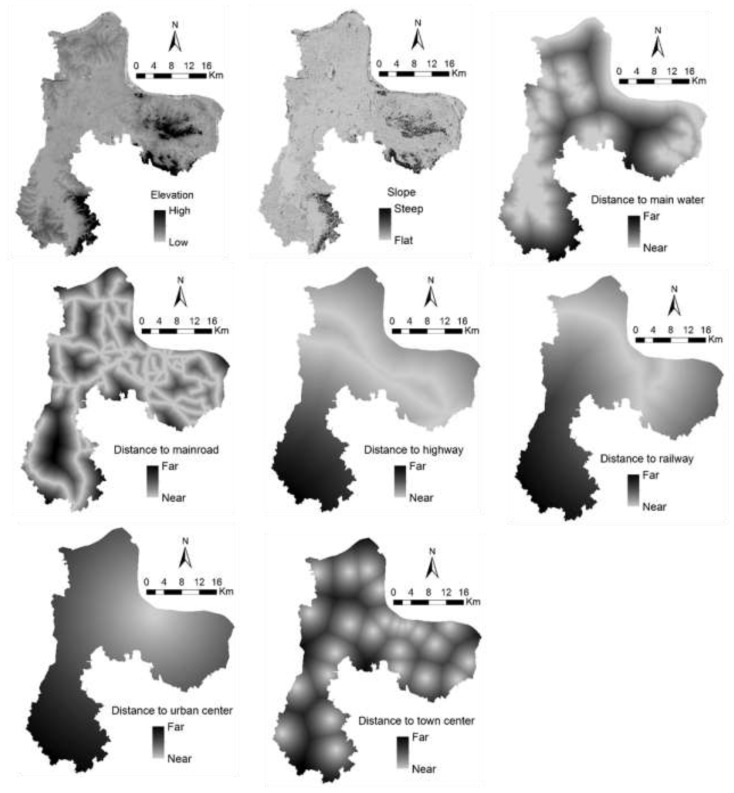
Maps of driving factors of land use and land cover change in the study region.

Drivers that we chose in this study include distance to highway, distance to railway, distance to major roads, distance to town centers, distance to urban centers, distance to large water bodies, elevation, and slope (see Figure 4 for detail). First, we conducted GIS-based overlay analysis on land cover data over two years, 1991 and 2004, to obtain the spatial distribution of land cover change in our study area for the calibration of the Markov-CA simulation model. Samples of land change types were generated through stratified random sampling (500 sampling points were used for each conversion type). Thus, sample data and driving factors are sent to the LSSVM model to produce the probability maps of alternative types of land cover change. The probability maps represent the spatial distribution of suitability for specific land cover change. Then, these land change suitability maps are input to the Markov-CA model. In this study, we used the Markov-CA module in IDRISI software (see [45]), which has a variety of applications [36,37,38]. Thus, we are able to simulate land cover change in our study area and conduct associated landscape pattern analysis as well as ecological risk analysis in particular, in a spatiotemporally explicit manner [46,47]. We used the year of 2004 as the initial time step and land transition matrix from 2004–2013 to determine the amounts of land transition. Three types of neighborhoods were examined: 1 × 1, 3 × 3, and 5 × 5. Model performance suggested the use of a 3 × 3 neighborhood for the Markov-CA model. We chose 6 months (a half year) as the temporal resolution of the model—*i.e.*, the number of iterations was set to 18 (*i.e.*, 9 years) for the 2004–2013 simulation.

#### 2.2.5. Scenario Analysis of Policy Intervention

Scenario analysis provides an approach that allows for the study of alternative futures of land systems through projections [48]. Based on the spatiotemporal simulation model, we designed four scenarios to examine future land change and associated landscape ecological risks in response to alternative policies (basic farmland protection, ecological conservation, and urban development) in our study region. Scenario 1 represents status quo, assuming the contribution of drivers remains unchanged over time. Scenario 2 is designed for the protection of farmlands. At present, our study region is planning for the identification and determination of permanent farmlands. Once a farmland is determined to be permanent, this land will not be allowed for any conversion. The total area of farmlands, therefore, will not decrease. Based on this, in Scenario 2, we fixed the location of farmlands that are already planned, and the total area of farmlands is not less than that in 2013. We used scenario 3 for the purpose of ecological conservation. According to the plan for the ecological conservation of Ezhou City, we increased the suitability of forests and water bodies by 20% for the first grade ecological conservation area, and by 10% for the second grade. Also, the area of water bodies and the area of forests are not less than those in 2013. Scenario 4 was designed to study prioritization on meeting land requirements for built-ups by adjusting the development probability of built-up lands 20% higher. For each scenario, we ran the Markov-CA simulation model to generate land cover patterns in 2015, 2020, 2025, and 2030. We then applied landscape pattern analysis and landscape ecological risk analysis to these simulated land cover patterns so as to evaluate possible future alternatives in response to policy intervention.

## 3. Results and Discussion

### 3.1. Results

**Figure 5:**
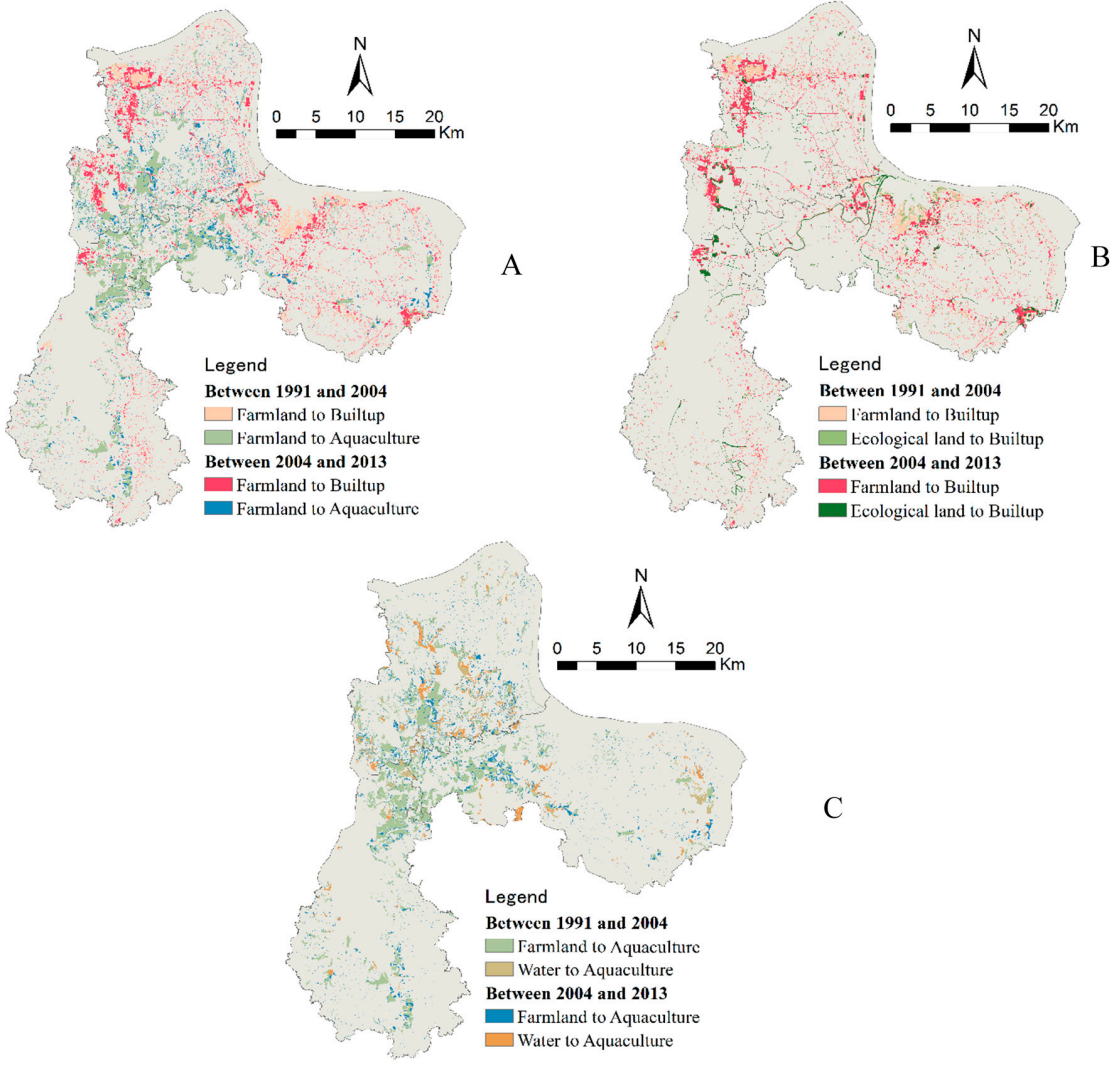
Spatial patterns of land conversion ((**A**) conversion from farmlands; (**B**) conversion to built-up; (**C**) conversion to aquaculture).

**Figure 6:**
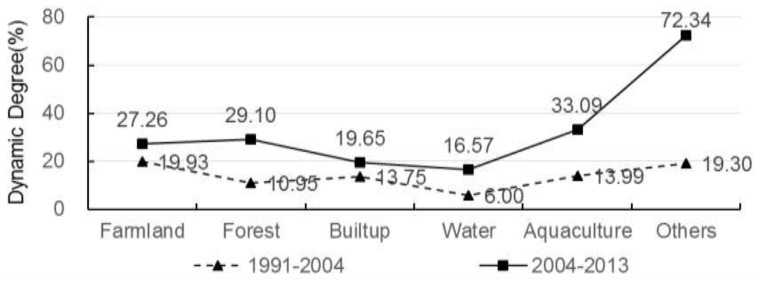
Dynamic degree index of land cover change.

Table 1 and Figure 5 report land cover change from 1991 to 2013 in our study area. It can be observed that farmland, water bodies, built-up land, and aquaculture lands dominated the initial stage of land cover patterns. During the period of 1991 to 2013, our study area experienced drastic land cover change. Figure 6 depicts the results of dynamic degree index for 1991–2004 and 2004–2013. Table 2 and Table 3 show results of land transition matrices for 1991–2004 and 2004 to 2013. It can be generally observed that land transition from 2004–2013 are more intensive than that from 1991–2004.

**Table 1:** Summary of land cover types between 1991 and 2013 (area unit: hectares).

Time	Farmland	Forest	Built-up	Water	Aquaculture	Others
Year 1991	87,858.27	8974.62	13,646.88	34,832.97	7376.94	8740.89
Percent	54.42%	5.56%	8.45%	21.58%	4.57%	5.41%
Year 2004	71,604.36	9193.95	18,870.84	32,990.40	19,871.28	8899.74
Percent	44.36%	5.70%	11.69%	20.44%	12.31%	5.51%
Year 2013	61,694.55	14,535.99	27,503.01	28,726.02	21,615.57	7355.43
Percent	38.22%	9.00%	17.04%	17.79%	13.39%	4.56%

**Table 2:** Land use transition matrix between 1991 and 2004 in the study area (unit: hectares).

	2004	Farmland	Forest	Built-up	Water	Aquaculture	Others
1991							
Farmland		70,348.32	425.07	5256.45	89.01	11,248.29	491.13
Forest		141.66	7992.18	400.86	1.62	120.96	317.34
Built-up		684.00	67.50	11,769.93	58.05	336.15	731.25
Water		152.19	96.66	178.65	32,744.34	1412.46	248.67
Aquaculture		158.49	11.97	713.52	90.45	6344.64	57.87
Others		119.70	600.57	551.43	6.93	408.78	7053.48
Net gain/loss		−16,253.9	219.33	5223.96	−1842.57	12,494.34	158.85

**Table 3:** Land use transition matrix between 2004 and 2013 in the study area (unit: hectares).

	2013	Farmland	Forest	Built-up	Water	Aquaculture	Others
2004							
Farmland		52,086.15	4495.41	7686.72	321.12	4587.84	2427.12
Forest		1307.52	6518.97	459.36	17.10	78.75	812.25
Built-up		2025.18	635.40	15,162.48	51.21	430.92	565.65
Water		965.34	112.59	1102.41	27,523.62	2513.07	773.37
Aquaculture		3625.65	117.72	1809.45	707.49	13,295.52	315.45
Others		1684.71	2655.90	1282.59	105.48	709.47	2461.59
Net gain/loss		−7901.55	7034.58	10,463.58	−886.23	7287.75	3206.43

Table 4 reports the results of landscape metrics for the years of 1991, 2004, and 2013. As we can see, contagion and patch density at the landscape level exhibit a decreasing pattern while splitting index and Shannon’s diversity index increase over time. Further, Appendix Table A1 shows results of class-level landscape metrics (for each land cover type). We computed splitting index and patch density for each town in Ezhou City over time (see Appendix Table A2).

**Table 4:** Landscape metrics in the study region over time (CONTAG: contagion; PAFRAC: perimeter-area fractal dimension; SPLIT: splitting index; SHDI: Shannon’s diversity index; PD: patch density).

Year	CONTAG	PAFRAC	SPLIT	SHDI	PD
1991	49.5346	1.3711	10.6097	1.3304	13.9540
2004	42.9412	1.3637	18.4742	1.5168	13.6851
2013	40.3263	1.3920	37.7513	1.6030	11.4966

**Figure 7:**
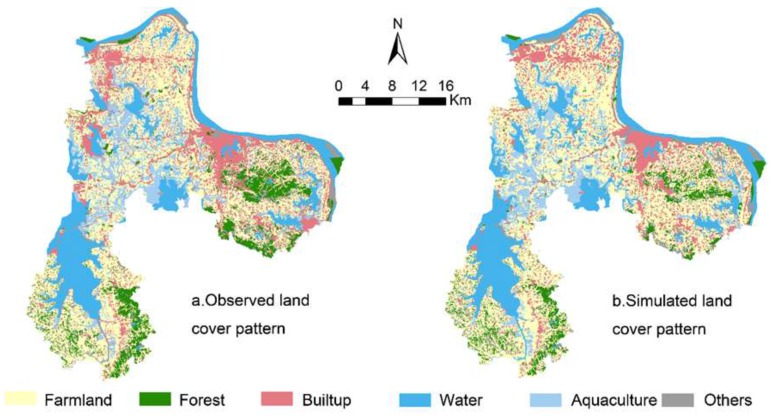
Spatial patterns of empirical and simulated land cover patterns of Ezhou City in 2013.

Based on the Markov-CA model, we obtained simulated land cover patterns in 2013 for our study area (see Figure 7). Through comparison between simulated land cover patterns and the observed one, we calculated model accuracy metrics, including overall model accuracy (percentage of correct match; see [49]) and Kappa coefficient (see [50]). Through visual inspection, we can see that the spatial patterns of simulated and observed land cover patterns match well. Appendix Table A3 shows results of model accuracy. The overall model accuracy is 67% and the Kappa coefficient is 0.53, showing a reasonably good agreement between simulated and empirical data. Thus, this model is acceptable for the spatiotemporal simulation of future land cover change. Then, we ran the simulation model for the four scenarios until 2030.

**Figure 8:**
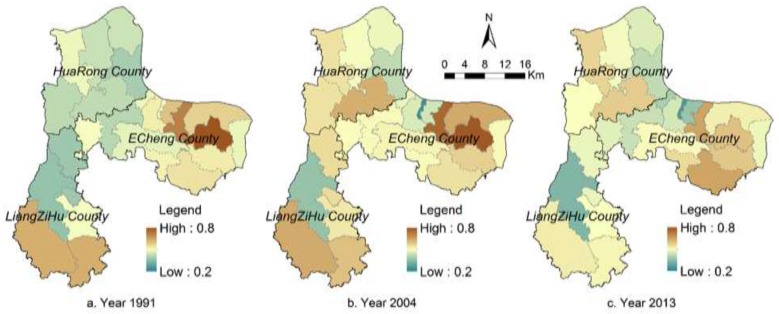
Maps of landscape ecological risk in the study region for year 1991, 2004, and 2013.

**Figure 9:**
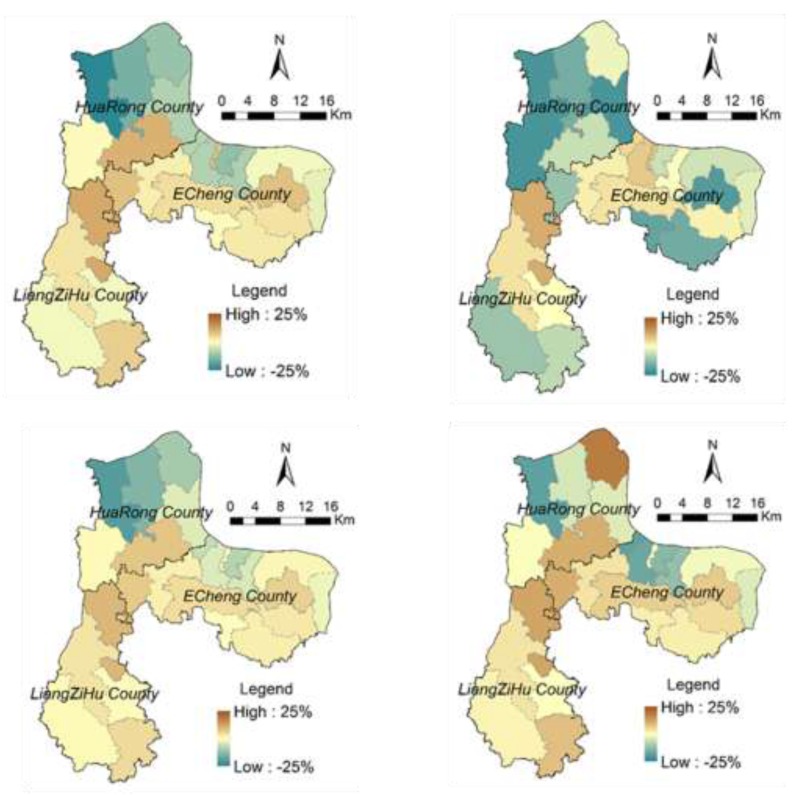
Spatial patterns of change ratios in town-level ecological risks in 2030 for different scenarios (with respect to 2013).

**Figure 10:**
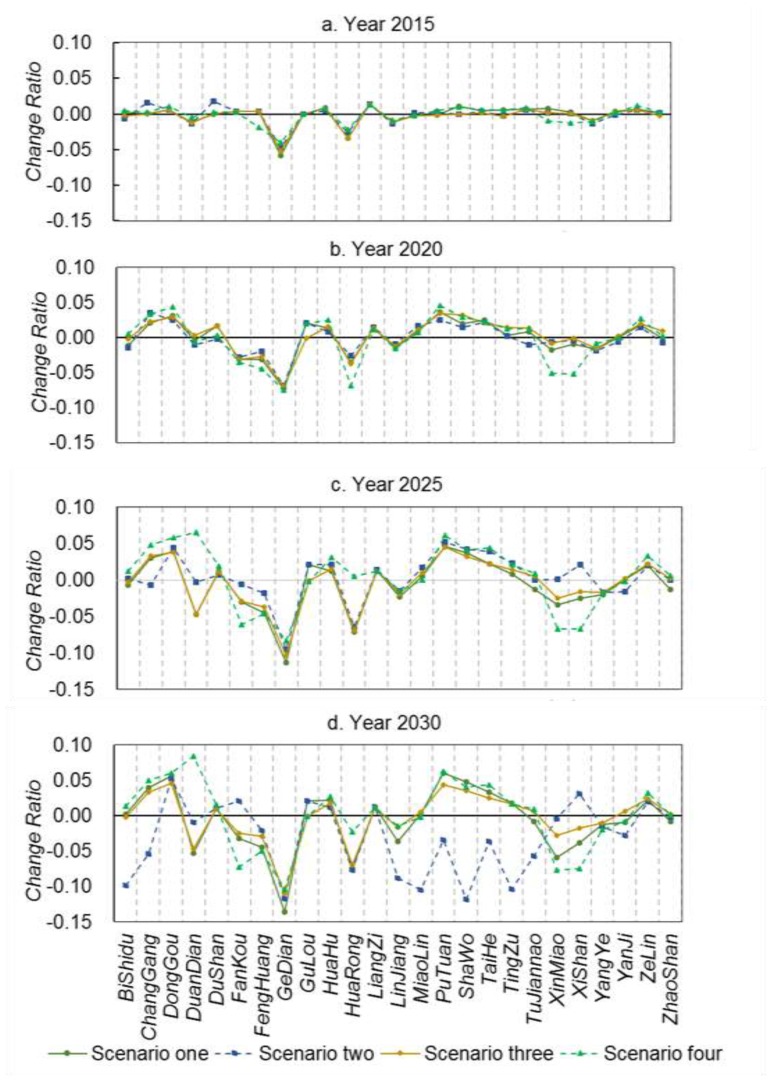
Temporal change of landscape ecological risks at the town level for different scenarios (with respect to 2013).

Figure 8 illustrates the spatial distribution of landscape ecological risks at the town level in our study region for the years of 1991, 2004, and 2013. The averaged town-level landscape ecological risk of the study region is 0.51 in 1991, 0.53 in 2004, and 0.49 in 2013, at a medium level of ecological risk. For the four scenarios with respect to alternative policy intervention, averaged landscape ecological risks of our study region from 2015 to 2030 remain at a medium level (0.46–0.49). Landscape ecological risks at the town level are spatiotemporally heterogeneous for the four scenarios used in this study, and changing patterns are different among these towns (see Figure 9 and Figure 10).

### 3.2. Discussion

#### 3.2.1. Overall Characteristics of Historic Land Cover Change

Our study region from 1991 to 2013 experienced substantial land cover change (see Table 1 and Figure 5), which led to severe loss of farmlands, rapid increase in built-up lands and aquaculture water bodies. First, while farmland is the dominant land cover type in our study area, the total area of farmland tends to decrease over time. The proportion of farmlands decreases from 54% in 1991 to 38% in 2013, corresponding to a loss of 26,163 hectares (30%). From the spatial distribution of farmland loss (see Figure 5a), we could observe that lost farmlands cluster in the middle of the study region (Hongxie Lake, BailiChangGang), in the downtown area of Ezhou City, and the Gedian Economic Development Zone. Second, in 1991, the percentage of built-up lands in our study region is only 8%. In 2013, this percentage reaches 17%, a net increase of 13,856 hectares (102% of net gain). In particular, built-up lands significantly increased between 2004 and 2013. Observed from the map of build-up land (Figure 5b), this change occurred in the northwestern part of the study region (close to Wuhan City), southeastern, and central city region of Ezhou City. Our field investigation revealed that an increase in built-up land generally falls within regions planned for development. Third, from 1991 to 2004, the proportion of aquaculture water bodies increased from the original 5% to 12% (169% net increase; *i.e.*, 12,494 hectares). From 2004–2013, aquaculture water bodies remain stable. This pattern can be attributed to the structural adjustment of regional agriculture and markets. Our study area is characterized by rich water resources. Between 1991 and 2004, driven by an increase in market revenue from aquaculture compared to farming, a large number of water bodies, including ponds, were converted for aquaculture (e.g., fish, shells, and lotus). After this period, as market revenue from aquaculture tends to be stable, the area of aquaculture water bodies remains almost unchanged. Increase in aquaculture water bodies mainly clustered in the middle of the study region (see Figure 5c), dominated by dense stream networks.

#### 3.2.2. Land Use Transition

The Markov transition matrices allow us to investigate conversions among specific land cover types, serving as a form of disturbance to landscape patterns. From Markov transition matrices in Table 2 and Table 3, we have the following findings.

*Intensive occupation of farmlands due to urban-rural expansion:* From 1991 to 2004, 5256 hectares (5.98%) of farmlands were converted to built-up lands. From 2004–2013, the area of farmlands converted to built-up lands is 7686 hectares (10.74%). From 1991 to 2013, the total area of farmlands converted to built-up lands is 12,843 hectares (16.72%). Thus, we could see that over different periods urban-rural expansion consumed large amounts of farmlands and this trend tends to be accelerated. This indicates that our study region, as a key part of the Greater Wuhan Metropolitan Region, has experienced rapid land development, imposing substantial influence on the socio-ecological environment. Ezhou is also a major region for grain in central China. The intensive occupation of farmlands will pose a severe threat for regional food security. Meanwhile, under the national policy of “requisition-compensation balance”, large amounts of rangelands or other lands were converted into low-quality farmland, which tends to degrade the environment of the study region.

*Increase in conversion of ecological lands into built-up*: From 1991 to 2004, about 1293 hectares of ecological land types (e.g., forest, water bodies, and aquaculture) were converted into built-up—*i.e.*, about 18.21% of land conversion. From 2004–2013, 3371 hectares of lands were converted into built-up (27.32% of land conversion). This means that ecological lands are becoming targets of urban-rural expansion, stimulated by strict farmland protection and large demands of built-up lands. This will potentially impose negative impacts on the development of ecologically representative regions in Ezhou City, which need new policies to prevent the continual loss of ecological lands.

*Frequent conversion between farmlands and aquaculture water bodies:* From 1991 to 2004, about 13,526 hectares of lands were converted to aquaculture, 83.16% from farmlands. Yet, only 158 hectares of aquaculture water bodies were converted into farmlands. From 2004–2013, the area of the lands converted into aquaculture water bodies is 8320 hectares (55.14% from farmlands). Under the policy of strict farmland protection and agriculture subsidy as well as a decrease in revenue from aquaculture markets, about 3625 hectares of aquaculture water bodies were converted back to farmlands. In total, the area of farmlands converted to aquaculture water bodies is 12,449 hectares and the area of aquaculture water bodies converted from farmlands is 3625 hectares. This shows a frequent conversion between farmlands and aquaculture water bodies. This conversion will adversely affect the landscape characteristics and quality of farmlands, which is neither good for landscape-level ecological security nor for the productivity of farmlands.

*Conversion of water bodies for aquaculture:* From 1991 to 2013, 3557 hectares of water bodies were converted for aquaculture purpose. However, only 294 hectares of aquaculture water bodies were converted back to regular water bodies. While conversion between regular water bodies and aquaculture ones is reversible, the large number of conversion tends to increase landscape vulnerability and its associated ecological risk.

*Impact of policies:* From 1991 to 2004, only 425 hectares of farmlands were converted into forests, yet this conversion reaches 4495 hectares from 2004 to 2013. This significant increase shows that the protection policies such as conversion of farmlands into forests for ecological restoration (Grain for Green program started in 1999) and requisition-compensation balance (started in 1997) are effective.

*Conversion of other land:* In this study, other land cover types include orchard, rangeland, wetlands, and other open space. From 1991 to 2004, about 1687 hectares of other lands were converted to forests, built-up, farmlands, and aquaculture. From 2004 to 2013, 6438 hectares of other lands were converted mainly to forests, built-up, farmlands, and aquaculture. This suggests that the conversion of other lands has been very intensive due to high demands for land resources in our study region. The excessive conversion of other lands (into built-up, farmlands, and aquaculture) may deteriorate environmental quality in our study area (due to pollution from, for example, untreated residential waste, pesticides, and fertilizers).

#### 3.2.3. Landscape Pattern Analysis of Land Cover Change

Overall landscape patterns of our study area tend to be more fragmented from 1991 to 2013 (see Table 4). The overall shape and characteristics of land patches became more complicated over time. From 1991 to 2013, farmlands tend to form into more separated parts, yet built-up lands became aggregated (see Appendix Table A1). Fragmented patterns of farmlands are attributed to the occupation of land developments in rural areas, which leads to the isolation of farmland patches. The patch density of forests increases from 1991 to 2004 but decreases from 2004 to 2013. The splitting index of forests decreases over time. This is mainly due to the national “Grain for Green” policy of converting farmlands back to forests between 2004 and 2013. This policy leads to the clustered pattern of forests. Patch density of water bodies exhibits a similar pattern to that of farmlands: increases first then decreases. However, the splitting index of water bodies tends to increase over time. From the land transition matrix (Table 2 and Table 3), we see that water bodies were mostly converted for aquaculture. Because large amounts of aquaculture water bodies were embedded in regular water bodies, patch density shows an increment from 1991 to 2004. After 2004, because of the adjustment of agriculture structure, small-sized aquaculture water bodies were converted into regular ones. This explains the decreasing pattern of patch density of regular water bodies after 2004. Correspondingly, patch density of aquaculture water bodies shows a decreasing pattern and the splitting index decreases over time.

Our results of town-level landscape metrics (see Appendix 2) suggest that the splitting index of most towns exhibited an increasing pattern (only the central city region and Gedian Economic Development zone show a decreasing pattern). Patch density of those towns with rapid (slow) land development from 1991 to 2004 decreases (increases) over time. From 2004 to 2013, patch density of most towns decreases due to the policy of land intensification, which encourages the aggregation of built-up and farmlands.

#### 3.2.4. Landscape Ecological Risks and Scenario Analysis

Most towns in the study region in 1991 are at the medium level of ecological risks (see Figure 8). Ecological risks of the eastern and southern parts of the study region are relatively high (close to 0.8). In 2004, ecological risks at the town level tend to increase (in particular, in the middle and north). In 2013, the eastern part of the study region experienced an increase in ecological risk, but in its mountainous area, ecological risk exhibits a decreasing pattern. In the Liangzi Lake region (also see Figure 1), ecological risks decrease substantially due to the establishment of a national-level ecologically representative region. Ecological risks in the Gedian Economic Zone and central city area increase. In general, from 1991 to 2013, ecological risks show an increasing pattern mainly due to the rapid urbanization process, leading to more landscape disturbance in our study region.

For the four scenarios, averaged town-level landscape ecological risks of our study region remain at a medium level (0.46–0.49) from 2015 to 2030. The landscape fragmentation index shows different patterns across scenarios. From 2015 to 2030, landscape fragmentation due to the protection policies of farmland or forests tends to be high at the early stage (patch density is 4.33 for Scenario 3 in 2015 and 3.80 for scenario 2 in 2020); the impact of landscape fragmentation due to the policy of urban planning tends to be high after 2025 (patch density is 3.71 and 3.56 for Scenario 4 in 2025 and 2030). At the level of land cover type, landscape fragmentation and splitting index for farmlands are the lowest in Scenario 2, yet these indices for built-up lands are higher than those in other scenarios. This shows that due to a strict farmland protection policy, urban development on those farmlands close to existing built-up lands are limited. In Scenario 3, due to the protection of forests and small change in spatial distribution of forested lands, landscape patterns of forest remain relatively stable. However, in Scenario 2, to ensure no decrease in the amounts of farmlands, conversion between forests and farmlands tends to be intensive, *i.e.*, an increase in conversion from forests and other lands to farmland, and an increase in converting low-quality farmland to forests. As a result, this leads to the clustering of forests, in line with the policy of forest conservation. Among these scenarios, landscape patterns of regular water bodies do not exhibit a substantial change. Landscape fragmentation for aquaculture water bodies tends to decrease but the splitting index shows an increasing pattern over time.

For towns of Gedian and Huarong (northwestern region; also see Figure 1), ecological risks show decreasing patterns over time, which can be attributed to continual increase in built-up lands under the influence of Wuhan Donghu High Tech Development Zone. For those towns rich in forests and water resources, landscape ecological risks tend to increase first then decrease (see Figure 10). This is because aquaculture water bodies and forests tend to be converted into farmlands, leading to an increase in landscape fragmentation. Yet, when farmlands become dominated, landscape fragmentation index and splitting index tend to decrease, which explains decrement in ecological risks. However, ecological risks in these towns in Scenario 3 exhibit an increasing pattern. This can be attributed to the conversion of forests and aquaculture water bodies (in second-level conservation zones) into farmlands, leading to increased landscape fragmentation.

## 4. Conclusions

Ezhou City is an important satellite city of the Greater Wuhan Metropolitan region in central China. This study region has experienced rapid LULCC driven by socio-economic development and national policies for urban development, ecological conservation, and farmland protection. In this study, we used a spatially explicit approach that is based on the integration of GIS and landscape pattern analysis to evaluate spatiotemporal characteristics of land cover patterns and associated landscape ecological risks. With support from land change simulation based on Markov-CA modeling, we conducted a scenario analysis for future landscape patterns in response to alternative policy intervention. The scenario analysis is pivotal to supporting policy making or adjustment of land use and ecological conservation. Our simulation outcomes and landscape ecological risk assessment for Ezhou City offer insight into the exploration of spatiotemporal complexity of land systems in study regions that are often ecologically vulnerable.

Specifically, results in this study show that from 1991 to 2013, farmlands, water bodies, built-up lands, and aquaculture water bodies remain as dominant land cover types. Yet, land cover patterns experience drastic change: severe loss of farmlands, substantial increase in aquaculture, and built-up lands. Urban-rural development has consumed significant amounts of farmland and ecological land. Meanwhile, frequent exchanges between farmlands and aquaculture water bodies potentially lead to negative impacts on land quality and landscape patterns. Under the policy of “requisition-compensation balance”, a significant number of other lands have been converted to farmlands, forests, and built-up lands. From 1991–2013, the overall landscape of our study region experienced substantial fragmentation: a decrease in landscape connectivity, and an increase in landscape diversity suggests intensive landscape disturbance from human activities. Further, landscape ecological risks in east and southeast Ezhou City are relatively high. An increase in landscape ecological risks in the southern part of Liangzi Lake was attributed to intensive land conversion. Results of scenario analyses in response to alternative policy interventions suggest that the planning of permanent basic farmlands in the study region holds potential in preventing landscape fragmentation and spontaneous urban expansion. More constraints on ecological conservation (e.g., regulation on development density) are needed for the protection of landscape-level environments in our study region.

## Appendix

**Table A1:** Landscape Metrics for Land Cover Types in the Study Region over Time.

Index	Time	Farmland	Forest	Built-up	Water	Aquaculture	Others
PD	1991	0.69	1.33	3.52	0.16	6.28	1.98
	2004	1.14	1.35	2.94	0.20	5.83	2.22
	2013	1.50	1.26	2.46	0.13	4.01	2.14
PAFRAC	1991	1.43	1.43	1.36	1.37	1.34	1.40
	2004	1.43	1.40	1.34	1.36	1.34	1.40
	2013	1.44	1.39	1.40	1.32	1.36	1.36
SPLIT	1991	13.40	24,697.46	9856.42	51.51	53,174.54	28,541.09
	2004	28.59	21,747.92	2530.97	54.28	3975.03	28,744.75
	2013	98.27	5125.79	108.04	156.29	2274.13	41,441.98

PD: patch density; PAFRAC: perimeter-area fractal dimension; SPLIT: splitting index; SHDI: Shannon’s diversity index.

**Table A2:** Landscape Metrics at the Town Level in the Study Region over Time.

County	Towns	Splitting Index			Patch Density		
		1991	2004	2013	1991	2004	2013
HuaRong	DuanDian	3.38	4.17	4.62	16.19	15.7	11.4
	GeDian	3.44	7.17	9.59	17.97	15.61	14.71
	HuaRong	2.96	4.16	8.59	16.88	16.32	14.81
	LinJiang	2.47	2.81	3.54	12.02	11.45	10.6
	MiaoLin	3.23	6.84	16.32	12.97	14.54	11.06
	PuTuan	2.24	4.47	42.78	10.87	14.84	14.27
LiangZi	DongGou	5.73	17.22	18.87	9.09	11.65	9.56
	LiangZi	1.43	1.44	1.45	3.26	3.31	2.64
	TaiHe	3.36	4.57	8.81	16.46	15.9	11.76
	TuJiannao	3.1	3.64	3.21	21.74	22.51	16.12
	ZhaoShan	2.35	2.95	3.43	16.62	17.24	14.37
ECheng	BiShidu	3.03	4.09	5.76	17.71	16.09	16.56
	ChangGang	3.1	7.02	11.68	10.41	9.29	10.41
	DuShan	5	11.65	10.85	6.37	7.17	6.71
	FanKou	6.68	5.06	3.06	7.96	8.55	6.49
	FengHuang	8.7	5.09	4.57	15.75	7.82	5.91
	GuLou	2.32	1.41	1.48	10.01	1.43	1.67
	HuaHu	5.31	5.95	12.58	13.72	14.23	12.92
	ShaWo	4.49	5.7	19.77	28.8	28.08	16.26
	TingZu	2.97	4.11	16.86	16.9	14.7	16.52
	XinMiao	3.81	14.5	9.69	29.13	29.06	23.48
	XiShan	4.54	4.69	4.08	14.51	10.52	13.4
	YangYe	4.43	6.06	9.77	9.14	8.94	6.9
	YanJi	4.09	8	10.32	21.55	19.49	13.29
	ZeLin	5.61	8.11	12.39	12.79	12.96	10.62

**Table A3:** Results of Model Accuracy (Area Units: Hectares; Accuracy Units: %).

Actual Simulated	Farmland	Forest	Built-up	Water	Aquaculture	Others	Total	Accuracy
Farmland	50,364	4524	6845	444	4479	2412	69,067	73
Forest	1353	6904	543	19	86	959	9864	70
Built-up	4134	718	16,573	63	1026	659	23,172	72
Water	871	91	958	2710	2241	601	7473	36
Aquaculture	3461	125	1597	812	13,168	382	19,544	67
Others	1495	2174	987	137	616	2342	7752	30
Total	61,678	14,536	27,503	4184	21,616	7355	136,872	67
